# Protein-based custom-designed molecular nanotraps for biomedical applications

**DOI:** 10.3762/bjnano.17.47

**Published:** 2026-05-21

**Authors:** Devid Maniglio, Alice Marinangeli, Alessandra Maria Bossi

**Affiliations:** 1 BIOtech Center for Biomedical Technologies, Department of Industrial Engineering, University of Trento, Via delle Regole, Trento, Italy; 2 Department of Biotechnology, University of Verona, Strada Le Grazie 15, 37134 Verona, Italy

**Keywords:** bioMIPs, gelatin methacryloyl (GelMA), meta-biomaterials, molecularly imprinted polymers, natural polymers silk fibroin, SilMA

## Abstract

The development of highly selective nanoscaled platforms for molecular recognition has gained significant attention in nanomedicine. Recently, we pioneered a versatile methodology for fabricating custom-designed molecular nanotraps using naturally derived polymers, by relying on protein building blocks and employing the molecular imprinting technique to confer selectivity toward specific molecular targets. With a portfolio of protein-based units that includes cross-linkable silk fibroin and gelatin, the approach enables tailored nanostructures capable of efficient and selective recognition towards molecular targets of clinical relevance. Envisaged applications span from targeting relevant biomarkers for therapeutics, diagnostics, and in situ sensing to building high-complexity order meta-biomaterials.

## Introduction

Molecular recognition is a cornerstone in biomedical nanotechnology; it is pivotal to applications ranging from drug delivery to sequestering and to sensing. Where traditional affinity systems, such as antibodies, fall short for inherent limitations such as limited stability, high costs, or inadequate selectivity, biomimetics have surged as potent alternatives [1]. A versatile way to form biomimetics is the molecular imprinting of polymers (MIP), which has emerged as a promising technique to create synthetic binding sites within polymer matrices that closely mimic natural recognition entities [2]. MIP technology is a strategy specifically designed to mold selective binding sites in a material through a template-assisted synthesis [3]. The specific recognition is achieved through a polymer synthesis that occurs in the presence of the desired molecule, which acts as a template. As a result, binding cavities with stereochemical complementarity to the molecular targets are formed in the nascent material. Particular interest was triggered by the possibility of preparing nanosized MIPs, such as MIP nanoparticles, abbreviated either as MIP NPs or nanoMIPs [4]. The state of the art in the synthesis of nanoMIPs largely relies on acrylamides, acrylates, and methacrylates as building blocks, forming de facto plastic antibodies [4]. Plastic nanoMIPs perfectly fit in assays and sensing devices for their robustness and long-term stability, but raise concerns when intended for therapeutic uses and systemic administration. Overcoming adverse effects, allergic reactions, and toxic degradation by-products led us to conceptualize nanoMIPs in a radically new way. Recently, the original use of natural polymers, in particular of proteins, as building blocks for the synthesis of imprinted bioderived materials was proposed, referring to them as bioMIPs [5–6]. Imprinting with biopolymers, such as proteins, could revolutionize the field and, ultimately, shape a new class of therapeutics and medical devices. The bioMIPs, by being fully biocompatible and non-toxic, de facto meet all the essential requirements for any intended clinical or biomedical applications, while improving sustainability [7–8]. From the concept to the practical aspects, a key question is: How should the choice of the natural biopolymer building blocks for the synthesis of bioMIPs be made? The answer is deeply linked to the intended final application. Natural polymers, such as polysaccharides, proteins, oligonucleic acids, and terpenes, offer a large variety of physicochemical properties deriving from their chemical structures [8]. Overall, proteins stand out for great variability in amino acid sequences, which provides charged, hydrophobic, and hydrophilic functionalities for molecular recognition. Proteins possess secondary structures, such as alpha helices and beta sheets, which can be exploited to modulate the stiffness of the formed bioMIPs. Ultimately, choosing the protein building block among those already in use in tissue engineering and regenerative medicine, such as gelatin and silk fibroin, ensures biocompatibility, biodegradability and non-toxicity. Despite these potentials, to date, the use of proteins as building blocks for the preparation of bioMIPs has remained relatively underexplored, for several well-founded reasons. First, proteins are traditionally interpreted within the paradigm that function is intrinsically linked to folding. Accordingly, proteins are widely employed as self-contained recognition elements by virtue of their well-defined three-dimensional structures, with prominent examples including monoclonal antibodies that exert their therapeutic activity through specific binding to the target molecule [9]. Only recently, research has begun to explore proteins as components for the fabrication of nanostructures, such as nanocages and nanoparticles [10–11]. However, the vast majority of these architectures are constructed through the ordered assembly of folded protein units. In such systems, nanoparticles and higher-order structures arise from the self-assembly of proteins; they combine modularity with a high degree of structural precision and enable functionalities such as molecular recognition and cargo delivery, which have the potential for overcoming some challenges of drug delivery. In contrast, the concept of exploiting unfolded proteins for binding and targeting applications remains largely counterintuitive and therefore less explored. From a conventional perspective, the lack of a defined structure is often perceived as incompatible with selective recognition. However, insights from natural systems challenge this view. Unfolded proteins, particularly intrinsically disordered proteins (IDPs), are increasingly recognized as functionally active, often undergoing partial folding upon interaction with molecular partners [12]. These observations are reshaping the current understanding of protein structure–function relationships, opening the possibility of employing unfolded or disordered proteins as functional building blocks. In this emerging framework, functionality is no longer strictly dictated by a predefined fold; it may instead arise from disordered assemblies and the spatial proximity of protein chains within a three-dimensional network. From this perspective, disorder characterizes the individual building blocks, whereas structural organization emerges at the material level through entanglement and packing. The resulting constructs exhibit defined shapes and three-dimensional architectures, whose collective and emergent properties may be described within the framework of protein-based metamaterials. Furthermore, introducing controlled covalent cross-linking (e.g., via methacrylation) [5] is a key factor to “freeze” structural disorder while preserving local functional zones. The concept of non-folded proteins assembled to form functional structures lays at the basis of the bioMIPs. Among the proteins so far explored for preparing bioMIPs, there is gelatin, which is obtained from collagen, the most plentiful structural protein in animals that offers mechanical strength and supports tissues like skin, tendons, and bones. While collagen consists of triple-helix polypeptide chains, which are particularly rich in glycine, proline, and hydroxyproline, gelatin is a denatured and fragmented form of collagen produced through partial acidic or basic hydrolysis [13]. Gelatin is a water-soluble polymer that typically undergoes a temperature-dependent gelation, with the transition from a liquid to a hydrogel occurring between 30 and 40 °C, depending on the source. Gelatin is often functionalized with pendant double bonds, yielding gelatin methacryloyl (GelMA), and suitable for cross-linking, which enables various degrees of structural stiffening as required by specific tissue engineering applications [14]. Besides, gelatin and GelMA hydrogels exhibit enhanced biocompatibility, making them suitable for biomedical applications, cell scaffolds, wound dressings, and drug delivery [13].

Silk is another promising protein-based material used in tissue engineering [15]. The structure of silk consists of two intertwined proteins, fibroin and sericin, with silk fibroin (SF) being non-immunogenic and highly biocompatible. Structurally, SF adopts mostly random coil conformations when dissolved in water but spontaneously self-assembles into beta sheets during processing. This beta-sheet formation enhances its mechanical strength, stability, and controlled biodegradability [16]. Also, SF is often functionalized with pendant double bonds to enable cross-linking and is often referred to as SilMA [17]. SF and SilMA find a wide use as cell scaffolds for tissue engineering and biofabrication. To date, GelMA and SilMA building blocks have been explored for forming bioMIPs, enabling selective and specific targeting of clinically relevant molecules, such as hormones, cytokines, and protein biomarkers [5–6,8,18–19].

## Results and Discussion

**Figure 1:**
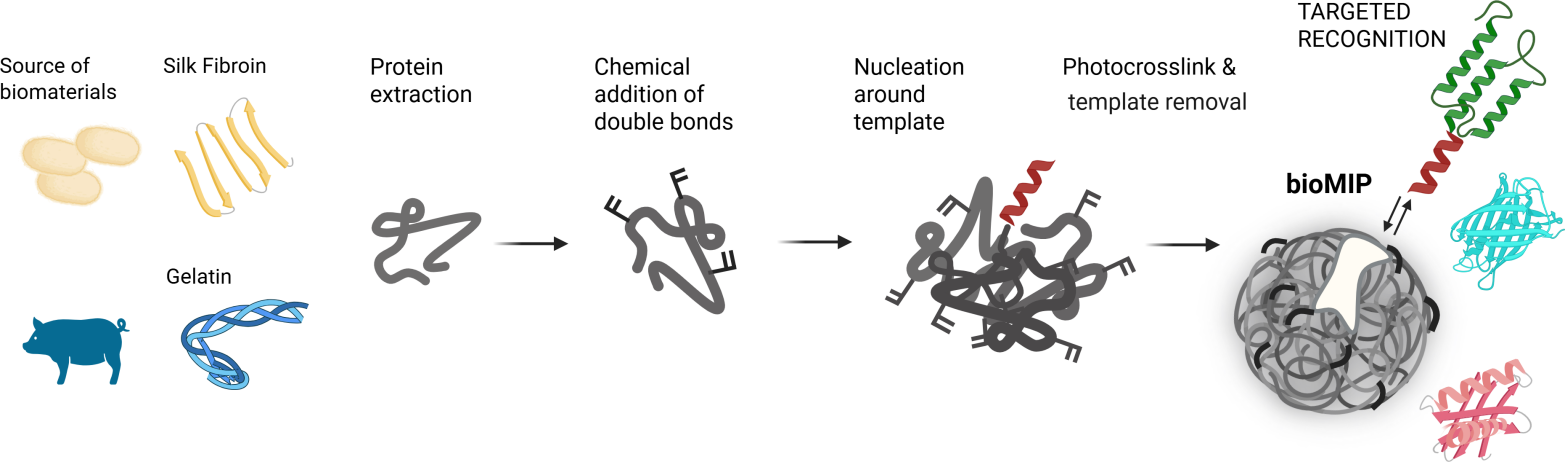
Among the possible sources of natural protein-based biopolymer building blocks for the imprinting process are silk fibroin and gelatin. The schematic steps for the synthesis of bioMIPs are depicted. The chosen protein building block is modified with pendant double bonds, such as GelMA and SilMA. Next these modified proteins are placed under conditions that trigger their nanoaggregation around the target molecule (i.e., peptide epitope in red). The so-formed nanoaggregate is stabilized by cross-linking, the template is removed by washing, and the bioMIP is ready to selectively rebind its target. Figure 1 was created in BioRender. Bossi, A. M. (2026) https://BioRender.com/20y7co2. This content is not subject to CC BY 4.0.

As described in Figure 1, with the aim to fabricate bioMIPs, gelatin and SF proteins were initially functionalized to make them cross-linkable via radical polymerization by introducing side-chain double bonds [14,17]. Next, SilMA or GelMA were dissolved at high dilutions (from 0.01 to 3 mg·mL^−1^) and allowed to spontaneously assemble into entangled disordered nanometric superstructures in the presence of the designed molecular target, so as to enable the imprinting process and form the specific binding sites [5–6]. Under highly diluted conditions, the process was observed to produce nanoscale aggregates of dimensions ranging from a few tens to a few hundreds of nanometers, and in the shape of nanoparticles. Next, photopolymerization of the double bonds had the purpose of fixing the final bioMIP conformation, preventing it from further structural modification, so as to keep in place the selective molecular recognition sites, conferred by the imprinting process (Figure 1). The whole process occurred in an aqueous environment, providing sustainable conditions. The surface response method [20–21] enabled the modeling of the optimal conditions for the synthesis of uniform bioMIPs, yielding nanoparticulates with a low polydispersity (PDI < 0.3). Investigations to elucidate the secondary structure of these protein-based bioMIPs by circular dichroism showed non-ordered protein assemblies.

It is worth considering that the selective ability of the bioMIPs is acquired upon the nanoscale aggregation of the protein building blocks around the target; hence, it is an emerging property of the biomaterials. For this, bioMIPs fit with the definition of meta-biomaterials. The bioMIPs do not require preserving the protein’s original fold to exert the recognition function; this simplifies manufacturing and hugely reduces costs while still providing effective and high-quality selective molecular recognition. Concerning the breadth of targets and the effectiveness of the molecular recognition, bioMIPs were prepared to recognize either entire proteins (e.g., human serum albumin [5]), or oligopeptides (the peptide hormone hepcidin [18]), or portions of a protein, that is, an epitope (C-terminus of interleukin-6 [6]). The tailored affinity towards the targets was remarkably high and comparable to that of natural antibodies, being estimated in the low nanomolar range. Selectivity for the target was proven, up to the level of discriminating scrambled sequences of the peptide targets [6]. Finally, GelMA bioMIPs were proven to behave like molecular nanotraps capable of sequestering specific proteins such as inflammatory markers, for example, interleukin-6 (IL-6), in physiologically relevant contexts. In fact, in vitro experiments using inflammatory cell models characterized by high levels of IL-6 showed that the addition of bioMIPs significantly reduced the levels of the pro-inflammatory cytokine, highlighting the potential therapeutic utility of bioMIPs.

## Future Directions

The preparation of bioMIP nanotraps represents a general strategy for fabricating protein-based molecularly selective meta-biomaterials. This innovative approach opens the way for developing next-generation biocompatible and biointegrable recognition materials, expanding towards nanodevices and nanostructures for targeted molecular recognition in nanomedicine and personalized therapy. The specific choice of protein polymers to be used as building blocks opens the possibility of exploiting the immense collection available, with millions of different protein candidates, benefiting from the high variability in charge and length and meeting the demand for custom-designing bioMIPs for any application. The scalability and environmental friendliness of this approach, together with the inherent biodegradability of the natural polymers, support their translational potential.

At a critical perspective, while the use of proteins for the preparation of bioMIPs could prove competitive with established strategies such as monoclonal antibodies [9], and comparable to emerging biomimetic systems such as polymeric telodendrimers [22–23] and to lipid-based therapeutics [24], careful consideration must be given to the potential limitations and challenges associated to this protein-based bioMIP approach; some shortfalls have been identified already in the context of folded protein systems. First, protein-based materials, depending on the biological source, are characterized by a wide variety in the physicochemical properties, given by the aminoacid compositions, secondary structure propensity, and the presence of isoforms. While the intrinsic complexity and variability of protein-based systems may facilitate the achievement of high complementarity with the intended molecular target, thus permitting a fine-tuning of the bioMIP function, it can also lead to substantial challenges in handling and in the reproducibility of bioMIPs. This may result in limited robustness and a degree of unpredictability in the performance of the resulting bioMIPs. In addition, the use of naturally derived biomaterials for intended biomedical applications requires access to controlled and reproducible protein sources. Achieving balance between cost-effectiveness and material quality remains nontrivial and may necessitate the use of recovered or waste-derived feedstocks, which must nonetheless meet stringent medical-grade certification requirements. Furthermore, the use of whole proteins introduces potential risks associated with immunogenicity due to the presence of short sequences capable of triggering immune responses. This constraint may significantly limit the range of suitable protein sources to prepare bioMIPs with the finality of clinical translation. Another aspect to consider is the occurrence of specific peptide motifs encoding biological functions (e.g., cell adhesion sequences such as RGD), which could lead to unintended biological interactions and off-target effects. Resulting from these considerations, the route to turn the first evidences on the potential of bioMIPs into consolidated nanomaterials for biomedical uses is still long and requires a deeper understanding of basic factors to govern not only bioMIP syntheses and recognition but also the fate of bioMIPs in complex contexts, including studies on the protein corona eventually formed on these bioMIPs.

Concerning the molecular recognition of bioMIPs and how to modulate it, these questions would require a rational approach to the choice of the type of protein building block, in dependence of the given molecular target and the selected clinical application. Research must explore a wider portfolio of natural materials for imprinting. Moreover, the interaction between any pair composed of a building block and a target molecule should be understood and modeled in thermodynamic and kinetic terms. Ideally, a tool to enable a rational choice of the protein building block for the bioMIP synthesis should be developed. Additionally, future endeavors must focus on in vivo efficacy, safety, long-term biostability, bioaccumulation, and biodegradation of by-products of the administered bioMIPs. Finally, the integration of bioMIPs into tissue replacements, or micro- and nanostructures for advanced diagnostics, meta-biomaterials, and therapeutic platforms is a field open to investigation.

## Data Availability

Data sharing is not applicable as no new data was generated or analyzed in this study.

## References

[1] Naik R R, Singamaneni S (2017). Chem Rev.

[2] BelBruno J J (2019). Chem Rev.

[3] Arshady R, Mosbach K (1981). Makromol Chem.

[4] Hoshino Y, Kodama T, Okahata Y, Shea K J (2008). J Am Chem Soc.

[5] Bossi A M, Bucciarelli A, Maniglio D (2021). ACS Appl Mater Interfaces.

[6] Bossi A M, Casella S, Stranieri C, Marinangeli A, Bucciarelli A, Fratta Pasini A M, Maniglio D (2025). Trends Biotechnol.

[7] Geissdoerfer M, Savaget P, Bocken N M P, Hultink E J (2017). J Cleaner Prod.

[8] Cowen T, Maniglio D, Bossi A M (2025). TrAC, Trends Anal Chem.

[9] Ebrahimi S B, Samanta D (2023). Nat Commun.

[10] Olshefsky A, Richardson C, Pun S H, King N P (2022). Bioconjugate Chem.

[11] Kaltbeitzel J, Wich P R (2023). Angew Chem, Int Ed.

[12] Miskei M, Horvath A, Vendruscolo M, Fuxreiter M (2020). J Mol Biol.

[13] Echave M C, Saenz del Burgo L, Pedraz J L, Orive G (2017). Curr Pharm Des.

[14] Yue K, Trujillo-de Santiago G, Alvarez M M, Tamayol A, Annabi N, Khademhosseini A (2015). Biomaterials.

[15] Vepari C, Kaplan D L (2007). Prog Polym Sci.

[16] Rockwood D N, Preda R C, Yücel T, Wang X, Lovett M L, Kaplan D L (2011). Nat Protoc.

[17] Kim S H, Yeon Y K, Lee J M, Chao J R, Lee Y J, Seo Y B, Sultan M T, Lee O J, Lee J S, Yoon S-i (2018). Nat Commun.

[18] Bossi A M, Maniglio D (2022). Microchim Acta.

[19] Maniglio D, Agostinacchio F, Bossi A M (2023). MRS Adv.

[20] Myers R H, Khuri A I, Carter W H (1989). Technometrics.

[21] Bucciarelli A, Muthukumar T, Kim J S, Kim W K, Quaranta A, Maniglio D, Khang G, Motta A (2019). ACS Biomater Sci Eng.

[22] Choi J, Moquin A, Bomal E, Na L, Maysinger D, Kakkar A (2017). Mol Pharmaceutics.

[23] Shi C, Guo D, Xiao K, Wang X, Wang L, Luo J (2015). Nat Commun.

[24] Brimacombe C A, Kulkarni J A, Cheng M H Y, An K, Witzigmann D, Cullis P R (2025). Mol Ther–Methods Clin Dev.

